# Wedge resection is an acceptable treatment option for radiologically low-grade lung cancer with solid predominance

**DOI:** 10.1093/icvts/ivac285

**Published:** 2023-01-09

**Authors:** Atsushi Kamigaichi, Takahiro Mimae, Norifumi Tsubokawa, Yoshihiro Miyata, Hiroyuki Adachi, Yoshihisa Shimada, Hiroyuki Ito, Norihiko Ikeda, Morihito Okada

**Affiliations:** Department of Surgical Oncology, Hiroshima University, Hiroshima, Japan; Department of Surgical Oncology, Hiroshima University, Hiroshima, Japan; Department of Surgical Oncology, Hiroshima University, Hiroshima, Japan; Department of Surgical Oncology, Hiroshima University, Hiroshima, Japan; Department of Thoracic Surgery, Kanagawa Cancer Center, Yokohama, Japan; Department of Surgery, Tokyo Medical University, Tokyo, Japan; Department of Thoracic Surgery, Kanagawa Cancer Center, Yokohama, Japan; Department of Surgery, Tokyo Medical University, Tokyo, Japan; Department of Surgical Oncology, Hiroshima University, Hiroshima, Japan

**Keywords:** Non-small-cell lung cancer, Wedge resection, Segmentectomy, Lobectomy, Maximum standardized uptake value, Ground-glass opacity

## Abstract

**OBJECTIVES:**

This study aimed to determine the clinical characteristics for predicting low-grade cancer in radiologically solid predominant non-small-cell lung cancer (NSCLC) and compare the survival outcomes of wedge resection with those of anatomical resection for patients with and without these characteristics.

**METHODS:**

Consecutive patients with clinical stages IA1–IA2 NSCLC showing radiologically solid predominance ≤2 cm at 3 institutions were retrospectively evaluated. Low-grade cancer was defined as the absence of nodal involvement and blood vessel, lymphatic and pleural invasion. The predictive criteria for low-grade cancer were established by multivariable analysis. The prognosis of wedge resection was compared with that of anatomical resection for patients who met the criteria, using the propensity score-matched analysis.

**RESULTS:**

Among 669 patients, multivariable analysis showed that ground-glass opacity (GGO) (*P *<* *0.001) on thin-section computed tomography and an increased maximum standardized uptake value on 18-fluoro-2-deoxyglucose positron emission tomography/computed tomography (*P *<* *0.001) were independent predictors of low-grade cancer. The predictive criteria were defined as GGO presence and maximum standardized uptake value ≤1.1 (specificity: 97.8%, sensitivity: 21.4%). In the propensity score-matched pairs (*n* = 189), overall survival (*P *=* *0.41) and relapse-free survival (*P *=* *0.18) were not significantly different between patients who underwent wedge resection and anatomical resection among those who fulfilled the criteria.

**CONCLUSIONS:**

The radiologic criteria for GGO and a low maximum standardized uptake value could predict low-grade cancer, even in solid-dominant NSCLC sized ≤2 cm. Wedge resection could be an acceptable surgical option for patients with radiologically predicted indolent NSCLC showing a solid-dominant appearance.

**Figure:**
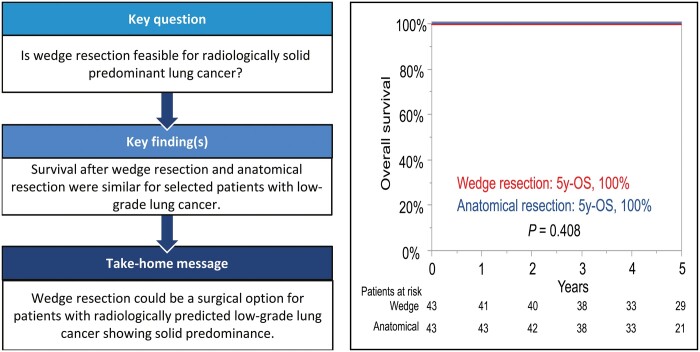

## INTRODUCTION

In 1995, the randomized prospective trial by the Lung Cancer Study Group reported poorer survival with a higher rate of recurrence in patients undergoing sublobar resection relative to patients undergoing lobectomy for early-stage non-small-cell lung cancer (NSCLC) [1]. Since then, the standard surgical procedure for NSCLC has been lobectomy with systematic nodal dissection or sampling. However, the development of radiological modalities, such as thin-section computed tomography (CT), has improved the accurate preoperative staging of NSCLC. A multicentre prospective study by the Japan Clinical Oncology Group (JCOG0201 trial) investigated radiologically diagnosed, non-invasive cancers based on the consolidation-to-tumour ratio (CTR) on thin-section CT [2]. In the exploratory analysis, at a cut-off value of 0.25, the specificity was the highest. Thus, the trial concluded that a pathological non-invasive cancer can be predicted by a CTR with a cut-off value of 0.25. A study after the trial reported that patients with clinical T1 tumours with CTR ≤0.5, classified as ‘low-grade’ lung cancer, have an excellent prognosis after complete surgical resection [3]. Based on the studies, wedge resection is an adequate oncological curative treatment option for patients with low-grade lung cancer with a wide ground-glass opacity (GGO) area [4, 5].

In contrast, tumours with CTR >0.5 are considered invasive and require anatomical pulmonary resection, such as segmentectomy or lobectomy, owing to the risk of pathologic invasiveness, including nodal involvement [6]. Recently, radiological modalities, such as 18-fluoro-2-deoxyglucose positron emission tomography/computed tomography (FDG-PET/CT) and thin-section CT, have been used widely to assess tumour invasiveness [7, 8]. In this study, we hypothesized that if low-grade lung cancer could be accurately identified by a combination of such modalities, curative resection could be achieved by wedge resection, even for patients with radiologically invasive lung cancer, i.e. tumours with CTR >0.5. Thus, this study aimed at the predictive identification of the subgroup of patients in whom the removal of lung cancer with CTR >0.5 by wedge resection would not constitute a loss of chance.

## MATERIALS AND METHODS

### Ethical statement

The Institutional Review Boards of the participating institutions approved this retrospective review of a prospective database and waived the requirement for informed consent from each patient (Hiroshima University Hospital: 13 June 2018; E-1216, Kanagawa Cancer Center: 28 February 2013; 24-KEN-54, Tokyo Medical University Hospital: 25 February 2015; SH2969).

### Patient population

We evaluated the medical records of 1129 consecutive patients with clinical stage IA1–IA2 lung cancer who underwent preoperative high-resolution CT, FDG-PET/CT and R0 curative surgery without induction therapy at 3 institutions between January 2010 and December 2019. Tumours were staged according to the TNM Classification of Malignant Tumours, 8th edition [9].

**Figure 1:**
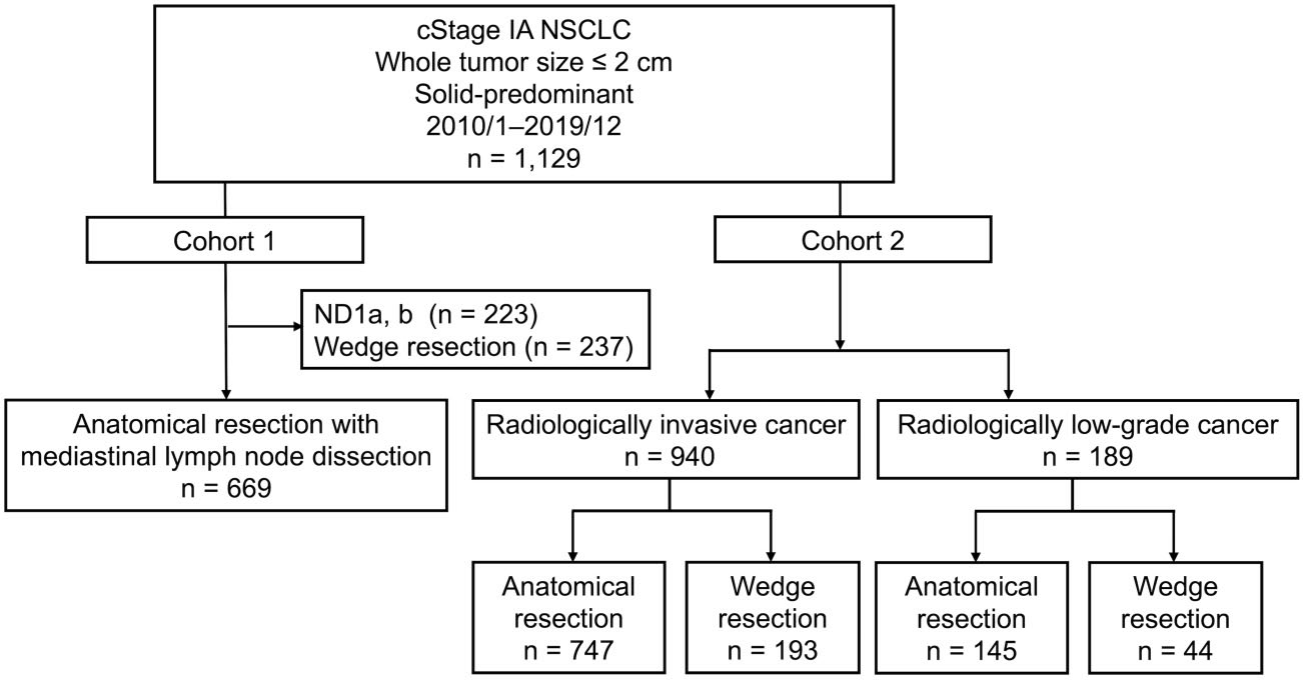
Flow chart of patient selection. In cohort 1, radiological predictors of pathologically confirmed low-grade lung cancer were identified. Subsequently, in cohort 2, the prognosis of wedge resection was compared with that of anatomical resection in patients with fulfilled (radiologically low-grade cancer) and unfulfilled (radiologically invasive cancer) criteria, defined in cohort 1.

We planned a two-stage, sequential cohort (Fig. 1).

#### Cohort 1: Establishing clinical predictive criteria for low-grade lung cancer

Patients who underwent anatomical resection with mediastinal nodal dissection were evaluated to identify the clinical predictors of pathologically confirmed low-grade lung cancer defined as the absence of nodal involvement and blood vessels, along with lymphatic and pleural invasions [3, 4, 10]. In Japan, hilar and mediastinal nodal evaluation is absent in wedge resection. Therefore, patients who underwent wedge resection or only hilar lymph node dissection were excluded due to inadequate assessment of nodal involvement.

#### Cohort 2: Comparing prognosis between wedge resection and anatomical resection according to the predictive criteria defined in cohort 1

The prognosis of wedge resection was compared with that of anatomical resection in patients fulfilling the low-grade tumour criteria defined in cohort 1 (radiologically determined low-grade lung cancer). A similar prognostic comparison was performed in patients who did not fulfil the low-grade tumour criteria. The types of procedures were decided by the surgeons and patients.

### Thin-section computed tomography and 18-fluoro-2-deoxyglucose positron emission tomography/computed tomography imaging

CT images were taken within 1 month before the surgery, and chest images were acquired using a 16-row multidetector CT. Thin-section images of the tumours were acquired using the following parameters: 120 kVp, 200 mA, section thickness 1–2 mm, resolution 512 × 512 pixels, scan duration 0.5–1.0 s and a high spatial reconstruction algorithm with a 20 cm field of view. The mediastinal and lung window settings were set at level 40 Hounsfield units (HU) and width 400 HU and level −600 HU and width 1600 HU, respectively. Surgeons and radiologists from each participating institution reviewed all CT images.

On FDG-PET/CT, an anthropomorphic body phantom conforming to the National Electrical Manufacturers Association standards was used to minimize the variability in the maximum standardized uptake value (SUV_max_), which could result from differences in preparation procedures, scan acquisition, image reconstruction and data analysis among the study centres [11].

### Pathologic evaluation

Lymphatic and blood vessel invasion was assessed by immunohistochemistry with D2-40, which stains the lymphatic ducts, and Verhoeff-van Gieson elastic staining of the elastic fibre of the vessels. Lymphatic and blood vessel invasion was defined as positive when the process of spreading through or penetration was detected as an extension of a malignant neoplasm. To evaluate pleural invasion, elastic tissue fibres were subjected to Verhoeff-van Gieson elastic staining. The pleural invasion was defined as positive if cancer had invaded the elastic layer, including invasion into the visceral pleural surface or neighbouring organs. Histologic examinations were performed by pathologists from each institution. The histologic type and pathological tumour size were reviewed according to the World Health Organization Classification of Tumours of the Lung, Pleura, Thymus and Heart, 2015 [10].

### Follow-up evaluation

Follow-up comprised physical examination and chest radiography every 3 months and a CT examination every 6 months for the first 2 years. Thereafter, physical examination and chest radiography were performed every 6 months, while CT was performed annually. Recurrence was determined based on the radiological features or histological evidence.

### Statistical analysis

Continuous variables are reported as medians and interquartile ranges (IQRs) and compared using the Wilcoxon rank-sum test. Categorical variables are presented as numbers (%) and compared using chi-squared or Fisher’s exact tests. We used McNemar’s test for categorical variables and paired *t*-test for continuous variables to analyse propensity-matched patient pairs.

To identify the clinical predictors of pathologically confirmed low-grade lung cancer, multivariable logistic regression analyses with a backward stepwise method were performed. The variables included age (≥65 or <65 years), sex (male or female), smoking history (ever or never), location (upper and middle lobe or lower lobe), solid tumour size (continuous), GGO (present or absent), SUV_max_ (continuous) and histological type (adenocarcinoma or others).

Receiver operating characteristic curves (ROC) of the SUV_max_ were generated to determine the criteria required to predict low-grade cancer. The point estimate of specificity ≥96% was determined as the cut-off value of SUV_max_ according to a previous study, which identified CT findings of non-invasive lung cancer [2].

Overall survival (OS) was defined as the time elapsed from surgery to death from any cause or censorship at the last follow-up before death. Recurrence-free survival (RFS) was defined as the time elapsed from surgery to recurrence, death from any cause or censorship at the last follow-up before death. Survival data between the wedge and anatomical resection groups were estimated using the Kaplan–Meier method and compared using the log-rank test. The propensity score was estimated using a logistic regression model, without the outcome information of survival, based on preoperative characteristics including age (≥65 or <65 years), sex (male or female), smoking history (ever or never), location (upper and middle lobe or lower lobe), solid tumour size (continuous), GGO (present or absent), SUV_max_ (continuous) and histological type (adenocarcinoma or others) as explanatory variables. Greedy matching with a calliper width of 0.20 of the standard deviation of the logit transformation for the estimated propensity score was applied. Propensity score matching at a 1:1 ratio was performed using the estimated propensity score. Standardized differences were calculated to investigate the balance of patient characteristics. The propensity score-matched analysis was performed in both patients fulfilling and not fulfilling low-grade tumour criteria.

Statistical analysis was performed using JMP version 14 (SAS Institute, Cary, NC, USA). *P*-values <0.05 were considered statistically significant without adjustment for multiple testing.

## RESULTS

### Identification of clinically predicted low-grade lung cancer: cohort 1

Supplementary Material, Table S1 summarizes the characteristics of the 669 patients in cohort 1. Pathologically confirmed low-grade lung cancer was observed in 398 (59.5%) patients who showed better OS than those with invasive lung cancer (*P *<* *0.001) (Supplementary Material, Fig. S1). The clinical predictors for detecting pathological low-grade lung cancer in solid-dominant NSCLC sized ≤2 cm included the presence of GGO [odds ratio (OR), 2.998; 95% confidence interval (CI), 1.856–4.843; *P *<* *0.001], SUV_max_ (OR, 0.716; 95% CI, 0.659–0.777; *P *<* *0.001) and male sex (OR, 0.589; 95% CI, 0.377–0.921; *P = *0.020) (Table 1).

**Table 1:** Uni- and multivariable logistic regression analyses for predictors of pathologically confirmed low-grade lung cancer

Variables	Univariable			Multivariable		
	OR	95% CI	*P*-Value	OR	95% CI	*P*-Value
Age (+1 year)						
<65	Ref.					
≥65	1.065	0.773–1.466	0.70			
Sex						
Female	Ref.			Ref.		
Male	0.430	0.311–0.594	<0.001	0.542	0.372–0.788	0.001
Smoking history						
Never	Ref.					
Ever	0.399	0.286–0.556	<0.001			
Tumour location						
Upper + middle lobe	Ref.					
Lower lobe	1.341	0.974–1.848	0.073			
Solid tumour size, +1.0 cm	0.224	0.139–0.361	<0.001			
Ground-glass opacity						
Absent	Ref.			Ref.		
Present	5.383	3.612–8.021	<0.001	2.811	1.815–4.354	<0.001
SUV_max_, +1.0	0.681	0.631–0.734	<0.001	0.720	0.667–0.777	<0.001
Histological type						
Others	Ref.					
Adenocarcinoma	3.020	2.037–4.477	<0.001			

CI: confidence interval; SUV_max_: maximum standardized uptake value.

**Figure 2:**
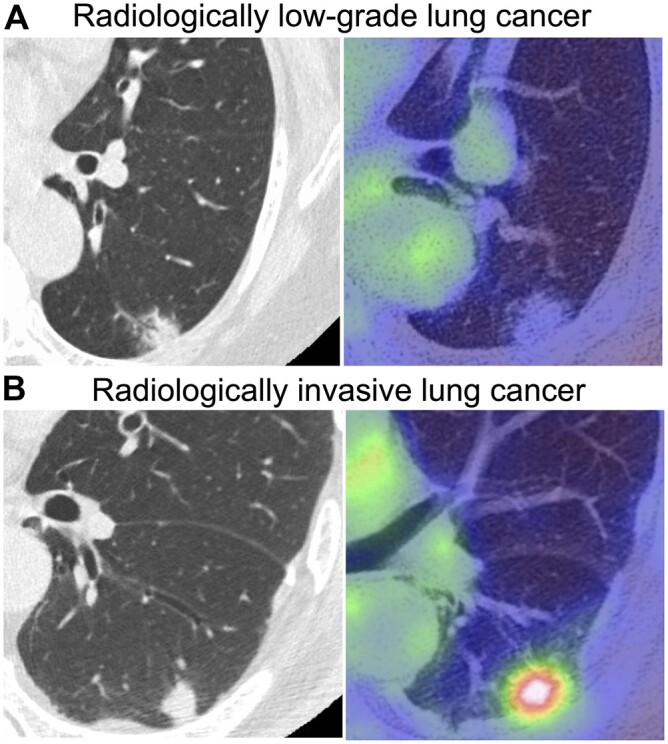
Examples of 2 solid predominant tumours sized ≤2 cm fulfilling (**A**) and not fulfilling (**B**) the low-grade tumour criteria (presence of ground-glass opacity and maximum standardized uptake value ≤1.1). (**A**) Whole tumour size, 1.9 cm; solid tumour size, 1.2 cm; maximum standardized uptake value, 0.5. (**B**) Whole tumour size, 1.2 cm; solid tumour size, 1.2 cm; maximum standardized uptake value, 5.5.

Based on the ROC analysis, the optimal cut-off value of the SUV_max_ was 1.1 (specificity, 96.3%; 95% CI, 93.3–98.2; sensitivity, 30.7%; 95% CI, 26.2–35.4; Supplementary Material, Fig. S2) (area under the curve of ROC, 0.813; 95% CI, 0.778–0.843). The criteria for clinically predicted low-grade lung cancer were the presence of GGO and SUV_max_ ≤1.1 (Fig. 2).

The specificity and sensitivity of the predictive criteria for the diagnosis of pathological invasive cancer were 97.8% (95% CI, 95.2–99.2) and 21.4% (95% CI, 17.4–25.7) (Table 2), respectively. Detailed pathological invasiveness of tumours between patients with fulfilled and unfulfilled low-grade tumour criteria are shown in Supplementary Material, Table S2. Significantly fewer patients with fulfilled low-grade tumour criteria had pathological invasiveness (*P *<* *0.001). Notably, no patients who fulfilled predictive criteria had nodal involvement.

**Table 2:** Relationship between radiological and pathological features

	Pathological diagnosis	
	Low grade^a^	Invasive
Clinical diagnosis		
Low grade^b^	85	6
Invasive	313	265

Specificity: 97.8% (95% CI: 95.2–99.2); sensitivity: 21.4% (95% CI: 17.4–25.7); positive predictive value: 93.4% (95% CI: 86.2–97.5); and negative predictive value: 45.8% (95% CI: 41.7–50.0).

a Pathological low-grade cancer was defined as the absence of nodal involvement and blood vessel, lymphatic and pleural invasion.

b Clinical criteria for the diagnosis of pathologically confirmed low-grade lung cancer was defined as the presence of ground-glass opacity component and maximum standardized uptake value ≤1.1.

CI: confidence interval.

### Prognosis of wedge resection compared to that of anatomical resection: cohort 2

The characteristics of 189 patients with fulfilled low-grade tumour criteria are presented in Supplementary Material, Table S3. The median follow-up duration was 61.6 months (IQR, 48.6–75.0 months). Distant recurrence occurred in 1 and 2 patients who underwent anatomical and wedge resections, respectively. Local recurrence did not occur among patients undergoing either surgical procedure. OS and RFS rates were not significantly different between the wedge resection (5-year OS, 100%; 5-year RFS, 94.5%; 95% CI, 80.3–98.7) and anatomical resection (5-year OS, 97.7%; 95% CI, 84.0–98.2; 5-year RFS, 97.0%; 95% CI, 82.3–97.3; *P *=* *0.89 and *P *=* *0.70, respectively) groups (Supplementary Material, Fig. S3A and B, respectively). Similar results were obtained when patients who underwent wedge resection were compared with those who underwent segmentectomy or lobectomy (Supplementary Material, Fig. S4A and B).

The characteristics of 940 patients with unfulfilled low-grade tumour criteria are shown in Supplementary Material, Table S4. The median follow-up duration was 54.4 months (IQR, 31.3–68.8 months). The OS and RFS rates were significantly worse in patients who underwent wedge resection (5-year OS, 73.3%; 95% CI, 65.1–80.2: 5-year RFS, 58.9%; 95% CI, 50.5–66.7) than those who underwent anatomical resection (5-year OS, 88.4%; 95% CI, 85.5–90.8; 5-year RFS, 81.3%; 95% CI, 78.0–84.2; *P *<* *0.001 and *P *<* *0.001, respectively) (Supplementary Material, Fig. S3C and D, respectively). A comparison of patients who underwent wedge resection and segmentectomy or lobectomy yielded similar results (Supplementary Material, Fig. S5A and B, respectively). Furthermore, the OS and RFS rates did not differ significantly between the 2 anatomical resection procedures (segmentectomy and lobectomy, *P *=* *0.55).

**Figure 3:**
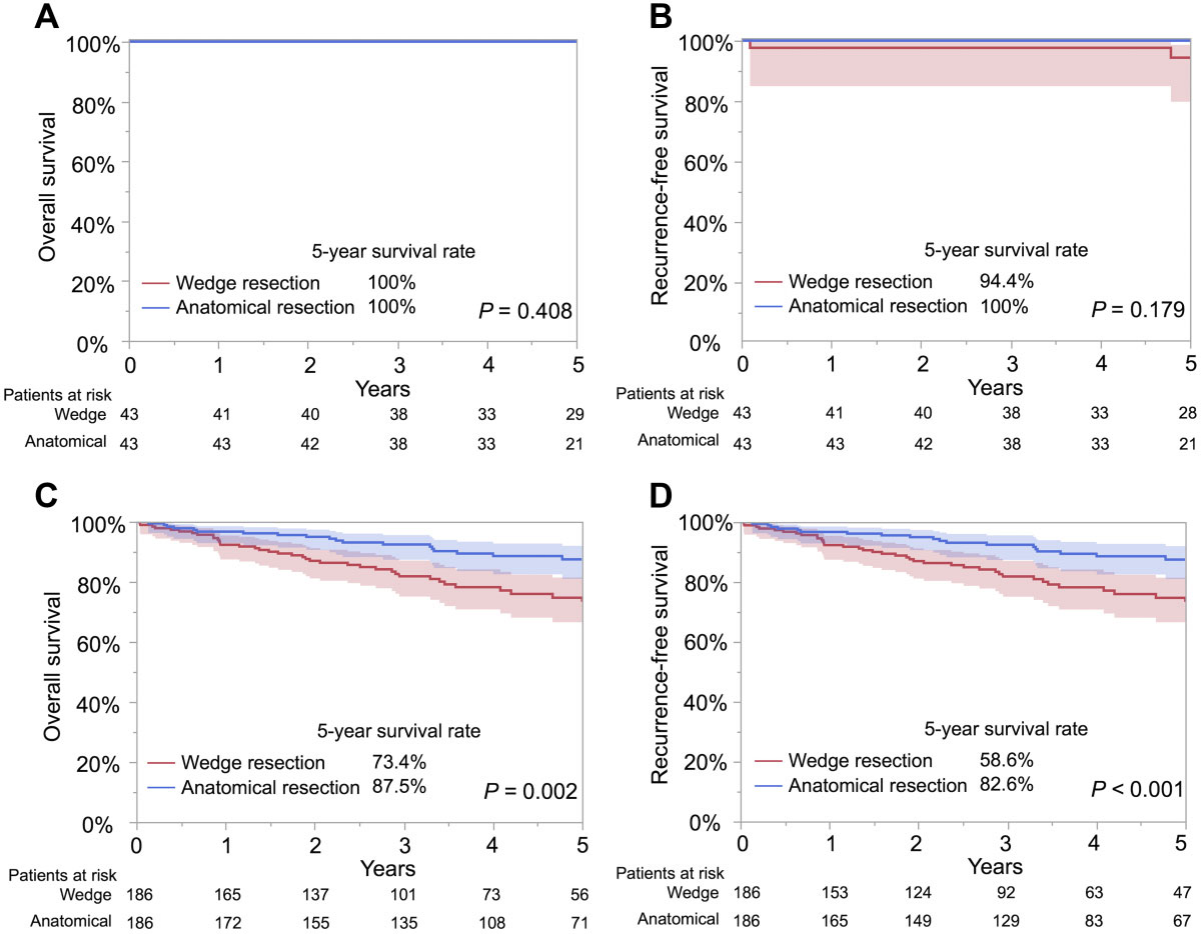
OS and RFS curves by the resection group in matched pairs with radiologically low-grade (presence of ground-glass opacity and maximum standardized uptake value ≤1.1) or invasive (absence of ground-glass opacity or maximum standardized uptake value >1.1) lung cancer. (**A**) Five-year OS rate was 100% after wedge resection and after anatomical resection (*P *=* *0.41) in patients with radiologically low-grade lung cancer. (**B**) Five-year RFS rate was 94.4% (95% CI, 79.7–98.6) after lobectomy and 100% after anatomical resection (*P *=* *0.179) in patients with radiologically low-grade lung cancer. (**C**) Five-year OS rate was 73.4% (95% CI, 64.9–80.4) with wedge resection and 87.5% (95% CI, 81.0–92.0) after anatomical resection (*P *=* *0.002) in patients with radiologically invasive lung cancer. (**D**) Five-year RFS rate was 58.6% (95% CI, 50.1–66.7) after wedge resection and 82.6% (95% CI, 75.7–87.9) after anatomical resection (*P* < 0.001) in patients with radiologically invasive lung cancer. CI: confidence interval; OS: overall survival; RFS: recurrence-free survival.

Propensity score matching of wedge resection and anatomical resection pairs (43 patients each in fulfilled low-grade tumour criteria; Table 3, 186 patients each in unfulfilled low-grade tumour criteria; Supplementary Material, Table S5) did not show significant differences in clinical factors. Similar to the unmatched cohort, OS (*P *=* *0.41) and RFS (*P *=* *0.18) were not significantly different between the wedge resection and anatomical resection groups in patients with fulfilled low-grade tumour criteria (Fig. 3A and B, respectively). However, the OS (*P *=* *0.002) and RFS (*P *<* *0.001) were significantly worse in patients who underwent wedge resection than in those who underwent anatomical resection in patients with unfulfilled low-grade tumour criteria (Fig. 3C and D, respectively).

**Table 3:** Characteristics of patients who underwent wedge or anatomical resection for radiologically low-grade non-small-cell lung cancer in propensity score-matched pairs

Variables	Wedge resection (*n* = 43)	Anatomical resection (*n* = 43)	*P*-Value	SD^a^
Age, *n* (%)				
>65 years	30 (69.8)	31 (72.1)	1.0	0.033
Sex, *n* (%)				
Male	28 (65.1)	28 (65.1)	1.0	0
Smoking history, *n* (%)				
Ever	14 (32.6)	16 (37.2)	0.821	0.133
Tumour location, *n* (%)			1.0	0.036
Upper + middle lobe	27 (62.8)	28 (65.1)		
Lower lobe	16 (37.2)	15 (34.9)		
Solid tumour size (cm), median [IQR]	0.84 [0.7–1.0]	0.85 [1.7–1.0]	0.434	0.036
SUV_max_, median [IQR]	0 [0–0.81]	0 [0–0.75]	0.701	−0.114
Clinical stage, *n* (%)			0.799	0.062
IA1	32 (74.4)	34 (79.1)		
IA2	11 (25.6)	9 (20.9)		
Histological type, *n* (%)			1.0	0
Adenocarcinoma	43 (100)	43 (100)		
Squamous cell carcinoma	0	0		
Others	0	0		
Histological subtypes of adenocarcinoma				
AIS/MIA/lepidic	16/4/12 (37.2/9.3/27.9)	10/7/7 (23.3/16.3/16.3)	0.134	
Papillary/acinar	3/3 (7.0/7.0)	12/4 (43.3/16.6)		
Solid/micropapillary	1/1 (2.3/2.3)	1/0 (13.0/1.6)		
IMA/others	1/2 (2.3/4.7)	2/0 (4.0/0.7)		
Pathological stage, *n* (%)				
0	11 (25.6)	6 (14.0)	0.242	
IA1/IA2/IA3	27/3/1 (62.8/7.0/2.3)	37/9/1 (62.8/20.9/2.3)		
IIB	1 (2.3)	0		
Lymph vessel invasion, *n* (%)	4 (9.3)	2 (4.7)	0.676	
Blood vessel invasion, *n* (%)	2 (4.7)	1 (2.3)	1.0	
Pleural invasion, *n* (%)	0	0	1.0	
Lymph node metastasis, *n* (%)	0	0	1.0	
Adjuvant therapy, *n* (%)	1 (2.3)	0	1.0	

a Standardized differences were provided for variables used for calculating the propensity score.

AIS: adenocarcinoma in situ; IMA: invasive mucinous adenocarcinoma; IQR: interquartile range; MIA: minimally invasive adenocarcinoma; SD: Standardized mean difference; SUV_max_: maximum standardized uptake value.

## DISCUSSION

Our results indicate that wedge resection is an acceptable procedure, even for NSCLC with a solid predominant appearance, if the low-grade tumour criteria are fulfilled. However, wedge resection is unsuitable for NSCLC cases who do not fulfil the criteria.

To date, the findings of thin-section CT imaging are the most important grading tool for tumour malignancy [2]. Tumours with a GGO component have a better prognosis with lower pathological invasiveness than those without (i.e. pure solid lung cancer) [12–15]. A favourable prognosis of tumours with a GGO component is observed even if it is small in tumours sized ≤2 cm [16]. Thus, the invasiveness of solid predominant NSCLC could be stratified by the presence of a GGO component.

The recent broad utilization of FDG-PET/CT permits precise clinical evaluation of tumour malignancy [7, 8]. Nevertheless, findings of low FDG accumulation are not sufficient to completely exclude the possibility of pathologically invasive cancer [17, 18]. The combination of a low SUV_max_ and GGO enabled the prediction of low-grade cancer with high accuracy, even in solid predominant lung cancer. Notably, no patients with fulfilled low-grade tumour criteria had nodal involvement, and they were considered good candidates for wedge resection.

Since the Lung Cancer Study Group trial, sublobar resections are often performed in frail and compromised patients at high risk from standard lobectomy [19–21]. Sublobar resection comprises 2 procedures—wedge resection and segmentectomy. Segmentectomy includes anatomical resection with a dissection of intrapulmonary and hilar lymph node dissection. A recent multicentre randomized study revealed a survival advantage of segmentectomy over lobectomy [6]. In contrast, wedge resection is a non-anatomical resection that cannot get close enough to intrapulmonary and hilar lymph nodes. Thus, wedge resection is an oncologically inferior procedure compared to anatomical segmentectomy. Several previous retrospective studies comparing wedge resection to segmentectomy for patients with early-stage NSCLC reported that wedge resection was associated with higher rates of local recurrence and inferior survival [18, 22–24]. In these studies, including a study on patients with exclusively NSCLC with a low SUV_max_, several patients with pathologically invasive lung cancer were included [18, 22–24]. Wedge resection is considered to be limited in its ability to achieve complete control over invasive lung cancers [18, 22–24]. On the other hand, in a multicentre prospective study (JCOG0804/WJOG4607L trial), sublobar resection, including 258 (82.2%) wedge resections and 56 (17.8%) segmentectomies, was associated with excellent survival without local recurrence (RFS, 99.7%) for GGO predominant NSCLC (whole tumour size ≤2 cm with CTR ≤0.25), indicating pathological non-invasive cancer [4]. Therefore, wedge resection could be a reasonable surgical option with excellent cancer control for low-grade cancer, even if tumours had a predominantly solid appearance.

Wedge resection is a less toxic surgical procedure than anatomical pulmonary resection. It confers advantages in terms of operative time, blood loss, length of hospitalization, duration of chest drainage and postoperative complications [19, 20, 25]. Moreover, wedge resection reduces the risk of other causes of death [20]. According to the JCOG0802/WJOG4607L trial, preservation of the lung parenchyma after surgery could provide a survival benefit, especially in death due to a second primary cancer or other diseases, even for patients without major comorbidities [6]. Thus, cancer control and benefits from the preservation of lung parenchyma should be considered while determining the extent of pulmonary resection for patients with early-stage NSCLC. Furthermore, stereotactic body radiotherapy or follow-up without resection, especially for low-grade lung cancer, may be suitable. Further studies are warranted.

### Limitations

This study has several limitations. First, it was a retrospective analysis of prospectively collected patient data, introducing a systematic bias that no inferential statistics can overcome [22, 26]. Moreover, the lack of preoperative data, like performance status, comorbidities, respiratory function and the preference of attending surgeons and patients in determining surgical procedures, might have led to selection biases. In addition, this study was based on a Japanese-based population cohort, and a validation study was not performed. Future validation studies in multiple countries and hospitals are warranted. Second, only 9.6% of the patient population fulfilled the low-grade tumour criteria, resulting in a small sample size. Third, lymph node dissection is mandatory following wedge resection in several regions, including Europe and North America, but not in Japan [6, 26]. Thus, the nodal staging was absent in patients undergoing wedge resection in this study. Nevertheless, accurate evaluation of lymph nodes at the pulmonary hilum or within the remaining lung lobe during wedge resection is challenging. Therefore, lung cancer without risk of nodal involvement, as identified in this study, is considered a good indication for wedge resection. Fourth, spread through air spaces, an important invasive factor in lung cancer, was not used in this study. Finally, the SUV_max_ cut-off value of 1.1 is based on a gauged mean standardized uptake value of individual standardized uptake values measured at each involved centre. Therefore, it can only be adopted in this study; even after harmonization, a more standardized value may be acquired in future by similar large observational studies that combine data from multiple centres.

## CONCLUSION

The radiologic criteria from the combined findings of high-resolution CT and FDG-PET/CT were valuable in predicting low-grade cancer, even in solid-dominant NSCLC sized ≤2 cm. Furthermore, wedge resection could be an acceptable surgical option for patients with radiologically predicted low-grade lung cancer.

## Supplementary Material

ivac285_Supplementary_Data

## Data Availability

The data underlying this study are available in this article and in the online supplementary material.

## ABBREVIATIONS

CI: Confidence interval
CT: Computed tomography
CTR: Consolidation-to-tumour ratio
FDG-PET/CT: 18-Fluoro-2-deoxyglucose positron emission tomography/computed tomography
GGO: Ground-glass opacity
HRCT: High-resolution computed tomography
HU: Hounsfield units
IQR: Interquartile range
NSCLC: Non-small-cell lung cancer
OR: Odds ratio
OS: Overall survival
RFS: Recurrence-free survival
SUV_max_: Maximum standardized uptake value
