# Supplementary material for: Wedge resection is an acceptable treatment option for radiologically low-grade lung cancer with solid predominance
Source: Interdiscip Cardiovasc Thorac Surg. 2023 Jan 9;36(1):ivac285. doi: 10.1093/icvts/ivac285 (PMC9931075; doi:10.1093/icvts/ivac285)
Supplement: ivac285_Supplementary_Data [file ivac285_supplementary_data.zip › Supple/Supplementary_Table_S5.docx]

| **Supplementary Table 5. Characteristics of patients in propensity score-matched pairs who underwent wedge or anatomical resection for radiologically invasive NSCLC.** | | | | |
| --- | --- | --- | --- | --- |
| Variables^a^ | Wedge resection | Anatomical resection |  |  |
|  | (n=186) | (n=186) | *P*-value | *SD* |
| Age, n (%) |  |  |  |  |
| >65y | 154 (82.8) | 158 (85.0) | 0.673 | 0.026 |
| Sex, n (%) |  |  |  |  |
| Male | 133 (71.5) | 118 (63.4) | 0.121 | 0.121 |
| Smoking history, n (%) |  |  |  |  |
| Ever | 139 (74.7) | 129 (69.4) | 0.298 | 0.074 |
| Tumour location, n (%) |  |  | 0.753 | 0.039 |
| Upper + Middle lobe | 104 (55.9) | 108 (58.1) |  |  |
| Lower lobe | 82 (44.1) | 78 (41.9) |  |  |
| Solid tumour size, cm | 1.3 [1.1–1.7] | 1.3 [1.1–1.5] | 0.206 | 0.149 |
| SUV_max_ | 2.3 [1.30–4.59] | 2.58 [1.40–4.05] | 0.749 | 0.062 |
| Clinical stage, n (%) |  |  | 0.594 | 0.033 |
| IA1 | 32 (17.2) | 37 (19.9) |  |  |
| IA2 | 154 (82.8) | 149 (80.1) |  |  |
| Histological type, n (%) |  |  | 0.774 | 0.051 |
| Adenocarcinoma | 114 (61.3) | 120 (64.5) |  |  |
| Squamous cell carcinoma | 47 (25.3) | 45 (24.2) |  |  |
| Others | 25 (13.4) | 21 (11.3) |  |  |
| Histological subtypes of adenocarcinoma |  |  | 0.368 |  |
| AIS/MIA/Lepidic | 11/5/19 (9.7/4.4/16.7) | 4/11/22 (3.3/9.2/18.3) |  |  |
| Papillary/Acinar | 35/18 (30.7/15.8) | 45/18 (37.5/15.0) |  |  |
| Solid/Micropapillary | 17/3 (14.9/2.6) | 12/3 (10.0/2.5) |  |  |
| IMA/Others | 5/1 (4.4/0.9) | 5/0 (4.2/0) |  |  |
| Pathological Stage, n (%) |  |  | 0.128 |  |
| 0 | 8 (4.3) | 6 (3.2) |  |  |
| IA1/IA2/IA3/IB | 41/76/15/37  (22.0/40.9/8.1/19.9) | 54/80/11  (29.0/43.0/5.9) |  |  |
| IIA/IIB | 1/5 (0.54/2.7) | 0/11 (0/5.9) |  |  |
| IIIA | 3 (1.6) | 4 (2.2) |  |  |
| Lymph vessel invasion, n (%) | 37 (19.9) | 37 (19.9) | 1.0 |  |
| Blood vessel invasion, n (%) | 64 (34.4) | 47 (25.3) | 0.07 |  |
| Pleural invasion, n (%) | 44 (23.7) | 21 (11.3) | 0.003 |  |
| Lymph node metastasis, n (%) | 1 (0.54) | 11 (5.9) | 1.0 |  |
| Adjuvant therapy, n (%) | 9 (4.8) | 12 (6.5) | 0.654 |  |
| Abbreviations: AIS, adenocarcinoma in situ; IMA, invasive mucinous adenocarcinoma; IQR, interquartile range; MIA, minimally invasive adenocarcinoma; NSCLC, non-small cell lung cancer; SD, Standardized mean difference; SUV_max_, maximum standardized uptake value  *Standardized differences were provided for variables used for calculating the propensity score | | | | |
