# Supplementary material for: Wedge resection is an acceptable treatment option for radiologically low-grade lung cancer with solid predominance
Source: Interdiscip Cardiovasc Thorac Surg. 2023 Jan 9;36(1):ivac285. doi: 10.1093/icvts/ivac285 (PMC9931075; doi:10.1093/icvts/ivac285)
Supplement: ivac285_Supplementary_Data [file ivac285_supplementary_data.zip › Supple/Supplementary_Figure_legend.docx]

Supplementary Figure 1. **OS curves for patients with radiologically low-grade lung cancer (GGO and SUVmax ≤ 1.1) compared to those with radiologically invasive lung cancer (absence of GGO or SUVmax > 1.1).**

Supplementary Figure 2. **Receiver operating characteristic curves of SUVmax for pathologically confirmed indolent lung cancer.**

The optimal cutoff value was 1.1 (n = 669; AUC = 0.813; 95% confidence interval, 0.778–0.843; P < 0.001).

Abbreviations: SUVmax, maximum standardized uptake value; AUC, are under the curve

Supplementary Figure 3. **OS and RFS curves by resection group in unmatched pairs with radiologically low-grade (presence of GGO and SUV_max_ ≤1.1) or invasive (absence of GGO or SUV_max_ >1.1) lung cancer.**

(A) Five-year OS rate was 100% after wedge resection and 97.7% (95% CI, 93.0 to 99.2) after anatomical resection (*P*=0.89) in patients with radiologically low-grade lung cancer.

(B) Five-year RFS rate was 94.5% (95% CI, 80.3 to 98.7) after lobectomy and 97.0% (95% CI, 92.2 to 98.9) after anatomical resection (*P*=0.70) in patients with radiologically low-grade lung cancer.

(C) Five-year OS rate was 73.3% (95% CI, 65.1 to 80.2) with wedge resection and 88.4% (95% CI, 85.5–90.8) after anatomical resection (*P* < 0.001) in patients with radiologically invasive lung cancer.

(D) Five-year RFS rate was 58.9% (95% CI, 50.5 to 66.7) after wedge resection and 81.3% (95% CI, 78.0 to 84.2) after anatomical resection (*P* < 0.001) in patients with radiologically invasive lung cancer.

Abbreviations: CI, confidence interval; GGO, ground-glass opacity; OS, overall survival; RFS, recurrence-free survival; SUV_max_, maximum standardized uptake value

Supplementary Figure 4. **OS and RFS curves by resection group in patients with radiologically predicted indolent lung cancer (GGO and SUVmax ≤ 1.1).**

(A) Five-year OS was 100% with wedge resection, 94.4% (95% CI 84.0–98.2) with segmentectomy, and 100% with lobectomy.

(B) Five-year RFS was 94.5% (95% CI 80.3–98.7) with wedge resection, 92.8% (95% CI 82.3–97.3) with segmentectomy, and 100% with lobectomy.

Abbreviations: CI, confidence interval; GGO, ground-glass opacity; OS, overall survival; RFS, recurrence-free survival; SUVmax, maximum standardized uptake value

Supplementary Figure 5. **OS and RFS curves by resection group in patients with radiologically invasive lung cancer (absence of GGO or SUVmax > 1.1).**

(A) Five-year OS was 73.3% (95% CI 65.1–80.2) with wedge resection, 88.4% (95% CI 82.8–92.4) with segmentectomy, and 88.4% (95% CI 84.9–91.2) with lobectomy.

(B) Five-year RFS was 58.9% (95% CI 50.5–66.7) with wedge resection, 84.0% (95% CI 77.8–88.7) with segmentectomy, and 80.3% (95% CI 76.2–83.7) with lobectomy.

Abbreviations: CI, confidence interval; GGO, ground-glass opacity; OS, overall survival; RFS, recurrence-free survival; SUVmax, maximum standardized uptake value
