# Supplementary material for: Wedge resection is an acceptable treatment option for radiologically low-grade lung cancer with solid predominance
Source: Interdiscip Cardiovasc Thorac Surg. 2023 Jan 9;36(1):ivac285. doi: 10.1093/icvts/ivac285 (PMC9931075; doi:10.1093/icvts/ivac285)
Supplement: ivac285_Supplementary_Data [file ivac285_supplementary_data.zip › Supple/Supplementary_Table_S1.docx]

| **Supplementary Table 1. Characteristics of patients who underwent anatomical lung resection involving dissection of the hilar and mediastinal lymph nodes (Cohort 1)** | |
| --- | --- |
| Variables | Cohort 1 |
|  | (n=669) |
| Age, n (%) |  |
| ≥65y | 423 (63.2) |
| Sex, n (%) |  |
| Male | 379 (56.7) |
| Smoking history, n (%) |  |
| Ever | 402 (60.1) |
| Tumour location, n (%) |  |
| RUL/RML/RLL | 233/48/151 (34.8/7.2/22.6) |
| LUL/LLL | 131/106 (19.6/15.8) |
| Solid tumour size, cm | 1.4 [1.1–1.7] |
| Ground-glass opacity, n (%) |  |
| Present | 220 (32.9) |
| SUV_max_ | 2.4 [1.3–4.8] |
| Clinical stage, n (%) |  |
| IA1/IA2 | 112/557 (16.7/83.3) |
| Surgical procedure, n (%) |  |
| Segmentectomy | 146 (21.8) |
| Lobectomy | 523 (78.2) |
| Histological type, n (%) |  |
| Adenocarcinoma | 537 (80.3) |
| Squamous cell carcinoma | 52 (7.8) |
| Others | 80 (12.0) |
| Pathological Stage, n (%) |  |
| 0 | 16 (2.4) |
| IA1/IA2/IA3/IB | 177/265/42/83 (26.5/29.6/6.3/12.4) |
| IIA/IIB | 2/49(0.3/7.3) |
| IIIA/IIIB | 32/3(4.8/0.5) |
| Lymph vessel invasion, n (%) | 158 (23.6) |
| Blood vessel invasion, n (%) | 177 (26.5) |
| Pleural invasion, n (%) | 107 (16.0) |
| Lymph node metastasis, n (%) | 68 (10.2) |
| Adjuvant therapy, n (%) | 108 (16.1) |
| Abbreviations: IQR, Interquartile range; LLL, left lower lobe; LUL, left upper lobe; RLL, right lower lobe; RML, right middle lobe; RUL, right upper lobe | |
