# Supplementary material for: Wedge resection is an acceptable treatment option for radiologically low-grade lung cancer with solid predominance
Source: Interdiscip Cardiovasc Thorac Surg. 2023 Jan 9;36(1):ivac285. doi: 10.1093/icvts/ivac285 (PMC9931075; doi:10.1093/icvts/ivac285)
Supplement: ivac285_Supplementary_Data [file ivac285_supplementary_data.zip › Supple/Supplementary_Table_S2.docx]

| **Supplementary Table 2. Comparison of pathological invasive characteristics between those with radiologically predicted indolent lung cancer and others.** | | | |
| --- | --- | --- | --- |
|  | Tumours with GGO and SUV_max_ ≤ 1.1  (n=91) | Others  (n = 578) | *P*-value |
| ly | 5 (5.5) | 153 (26.5) | < 0.001 |
| v | 2 (2.2) | 175 (30.3) | < 0.001 |
| pl | 0 | 107 (18.5) | < 0.001 |
| N | 0 | 68 (11.8) | < 0.001 |

Abbreviations: GGO, ground-glass opacity; ly, lymphatic invasion; N, nodal metastasis; pl, pleural invasion; SUV_max_, maximum standardized uptake value; v, blood vessel invasion

Data are shown as number (%).
