# Supplementary material for: Wedge resection is an acceptable treatment option for radiologically low-grade lung cancer with solid predominance
Source: Interdiscip Cardiovasc Thorac Surg. 2023 Jan 9;36(1):ivac285. doi: 10.1093/icvts/ivac285 (PMC9931075; doi:10.1093/icvts/ivac285)
Supplement: ivac285_Supplementary_Data [file ivac285_supplementary_data.zip › Supple/Supplementary_Table_S3.docx]

| **Supplementary Table 3. Characteristics of patients who underwent wedge resection or anatomical resection for radiologically low-grade lung cancer** | | | |
| --- | --- | --- | --- |
| Variables^a^ | Wedge resection | Anatomical resection |  |
|  | (n=44) | (n=145) | *P*-value |
| Age, n (%) |  |  |  |
| > 65 y | 31 (70.5) | 85 (58.6) | 0.22 |
| Sex, n (%) |  |  |  |
| Male | 28 (63.6) | 92 (63.5) | 1.0 |
| Smoking history, n (%) |  |  |  |
| Ever | 15 (30.1) | 54 (37.2) | 0.86 |
| Tumour location, n (%) |  |  |  |
| RUL/RML/RLL | 14/3/5 (31.8/6.8/11.4) | 53/14/31 (36.6/9.7/21.4) | 0.27 |
| LUL/LLL | 10/12 (22.7/27.3) | 21/26 (14.5/17.9) |  |
| Solid tumour size, cm | 0.8 [0.7–1.0] | 1.0 [0.8–1.2] | 0.013 |
| SUV_max_ | 0 [0–0.8] | 0.7 [0–0.9] | 0.012 |
| Clinical stage, n (%) |  |  |  |
| IA1 | 33 (75.0) | 79 (54.5) | 0.022 |
| IA2 | 11 (25.0) | 66 (45.5) |  |
| Histological type, n (%) |  |  |  |
| Adenocarcinoma | 43 (97.7) | 145 (100) | 0.23 |
| Squamous cell carcinoma | 1 (2.3) | 0 |  |
| Histological subtypes of adenocarcinoma |  |  |  |
| AIS/MIA/Lepidic | 16/4/12 (37.2/9.3/27.9) | 27/24/31 (18.6/16.6/21.4) | <0.001 |
| Papillary/Acinar | 3/3 (7.0/7.0) | 42/12 (29.0/8.3) |  |
| Solid/Micropapillary | 1/1 (2.3/2.3) | 1/0 (0.7/0) |  |
| IMA/Others | 1/2 (2.3/4.7) | 8/0 (5.5/0) |  |
| Pathological Stage, n (%) |  |  |  |
| 0 | 11(25.0) | 20 (13.8) | 0.033 |
| IA1/IA2/IA3 | 27/4/1/ (61.4/9.1/2.3) | 80/37/7 (55.2/25.5/4.8) |  |
| IIA/IIB | 0/1 (0/2.3) | 1/0 (0.7/0) |  |
| Lymph vessel invasion, n (%) | 3 (6.8) | 6 (4.1) | 0.44 |
| Blood vessel invasion, n (%) | 2 (4.6) | 2 (1.4) | 0.23 |
| Pleural invasion, n (%) | 0 | 0 | 1.0 |
| Lymph node metastasis, n (%) | 0 | 0 | 1.0 |
| Adjuvant therapy, n (%) | 1 (2.3) | 3 (2.1) | 1.0 |
| Abbreviations: AIS, adenocarcinoma in situ; IMA, invasive mucinous adenocarcinoma; IQR, interquartile range; LLL, left lower lobe; LUL, left upper lobe; MIA, minimally invasive adenocarcinoma; RLL, right lower lobe; RML, right middle lobe; RUL, right upper lobe; SUV_max_, maximum standardized uptake value | | | |
