# Supplementary material for: Wedge resection is an acceptable treatment option for radiologically low-grade lung cancer with solid predominance
Source: Interdiscip Cardiovasc Thorac Surg. 2023 Jan 9;36(1):ivac285. doi: 10.1093/icvts/ivac285 (PMC9931075; doi:10.1093/icvts/ivac285)
Supplement: ivac285_Supplementary_Data [file ivac285_supplementary_data.zip › Supple/Supplementary_Table_S4.docx]

| **Supplementary Table 4. Characteristics of patients who underwent wedge or anatomical resection for radiologically invasive NSCLC.** | | | |
| --- | --- | --- | --- |
| Variables^a^ | Wedge resection | Anatomical resection |  |
|  | (n=193) | (n=747) | *P*-value |
| Age, n (%) |  |  |  |
| >65y | 161 (83.2) | 512 (68.5) | <0.001 |
| Sex, n (%) |  |  |  |
| Male | 53 (27.5) | 304 (40.7) | <0.001 |
| Smoking history, n (%) |  |  |  |
| Ever | 146 (75.9) | 481 (64.4) | 0.004 |
| Tumour location, n (%) |  |  |  |
| RUL/RML/RLL | 61/8/38 (31.6/4.2/19.7) | 233/58/156 (31.2/7.8/20.9) | 0.019 |
| LUL/LLL | 38/48 (19.7/24.9) | 184/116 (24.6/15.0) |  |
| Solid tumour size, cm | 1.3 [1.1–1.7] | 1.5 [1.2–1.7] | <0.001 |
| SUV_max_ | 2.2 [1.2–4.5] | 2.7 [1.6–5.2] | 0.001 |
| Clinical stage, n (%) |  |  |  |
| IA1 | 39 (20.2) | 97 (13.0) | 0.016 |
| IA2 | 154 (80.0) | 650 (87.0) |  |
| Histological type, n (%) |  |  |  |
| Adenocarcinoma | 114 (59.1) | 571 (76.4) | <0.001 |
| Squamous cell carcinoma | 51 (26.4) | 105 (14.1) |  |
| Others | 28 (14.5) | 71 (9.5) |  |
| Histological subtypes of adenocarcinoma |  |  |  |
| AIS/MIA/Lepidic | 16/4/12 (37.2/9.3/27.9) | 11/31/76 (1.9/5.4/13.3) | 0.003 |
| Papillary/Acinar | 3/3 (7.0/7.0) | 248/95 (43.3/16.6) |  |
| Solid/Micropapillary | 1/1 (2.3/2.3) | 74/9 (13.0/1.6) |  |
| IMA/Others | 1/2 (2.3/4.7) | 23/4 (4.0/0.7) |  |
| Pathological Stage, n (%) |  |  |  |
| 0 | 8(4.2) | 12 (1.6) | 0.017 |
| IA1/IA2/IA3/IB | 46/77/77/37 (23.8/39.9/7.8/19.2) | 180/314/43 (24.1/42.0/5.8) |  |
| IIA/IIB | 1/6 (0.5/3.1) | 1/65 (0.1/8.7) |  |
| IIIA/IIIB | 3/0 (1.6/0) | 32/3 (4.3/0.4) |  |
| Lymph vessel invasion, n (%) | 3 (6.8) | 2 (3.1) | 0.396 |
| Blood vessel invasion, n (%) | 2 (4.6) | 0 | 0.164 |
| Pleural invasion, n (%) | 0 | 0 | 1.0 |
| Lymph node metastasis, n (%) | 0 | 0 | 1.0 |
| Adjuvant therapy, n (%) | 1 (2.3) | 1 (1.6) | 1.0 |
| Abbreviations: AIS, adenocarcinoma in situ; IMA, invasive mucinous adenocarcinoma; IQR, interquartile range; LLL, left lower lobe; LUL, left upper lobe; MIA, minimally invasive adenocarcinoma; NSCLC, non-small cell lung cancer; RLL, right lower lobe. RML, right middle lobe; RUL, right upper lobe; SUV_max_, maximum standardized uptake value | | | |
