# Supplementary figures and images for: Wedge resection is an acceptable treatment option for radiologically low-grade lung cancer with solid predominance
Source: Interdiscip Cardiovasc Thorac Surg. 2023 Jan 9;36(1):ivac285. doi: 10.1093/icvts/ivac285 (PMC9931075; doi:10.1093/icvts/ivac285)

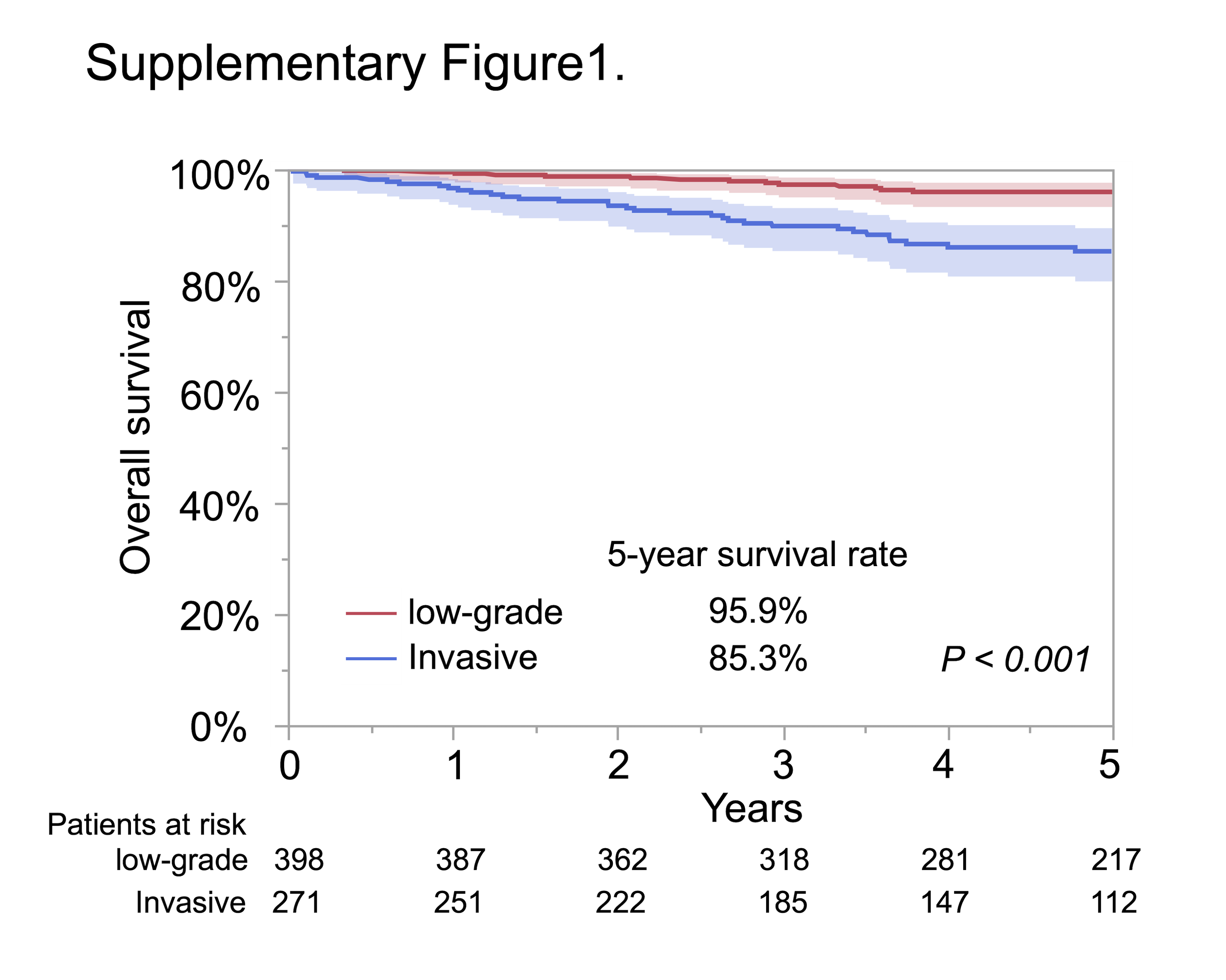

Supplement: ivac285_Supplementary_Data [file ivac285_supplementary_data.zip › Supple/Supplementary Figure 1.tiff]

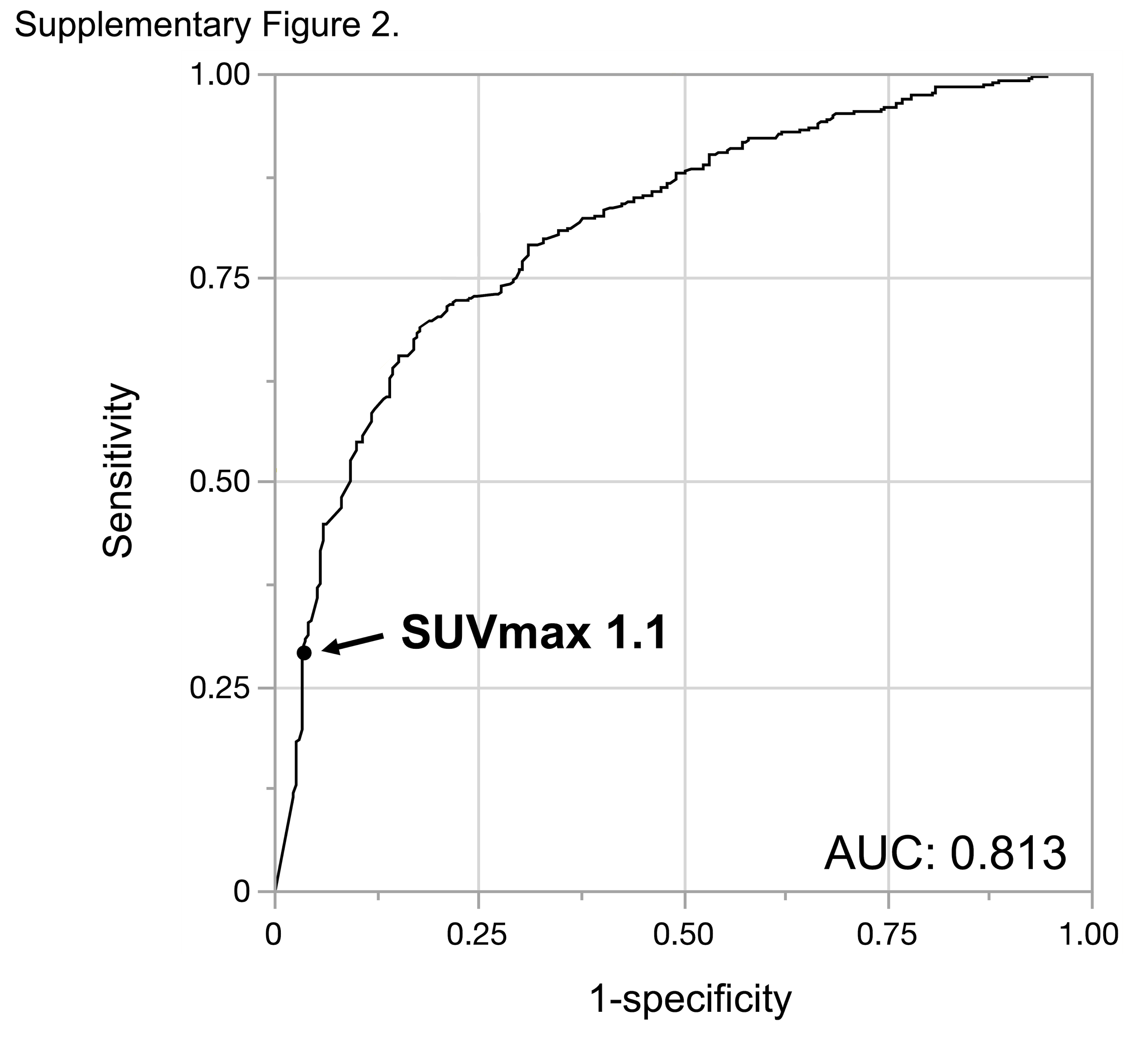

Supplement: ivac285_Supplementary_Data [file ivac285_supplementary_data.zip › Supple/Supplementary Figure 2.tiff]

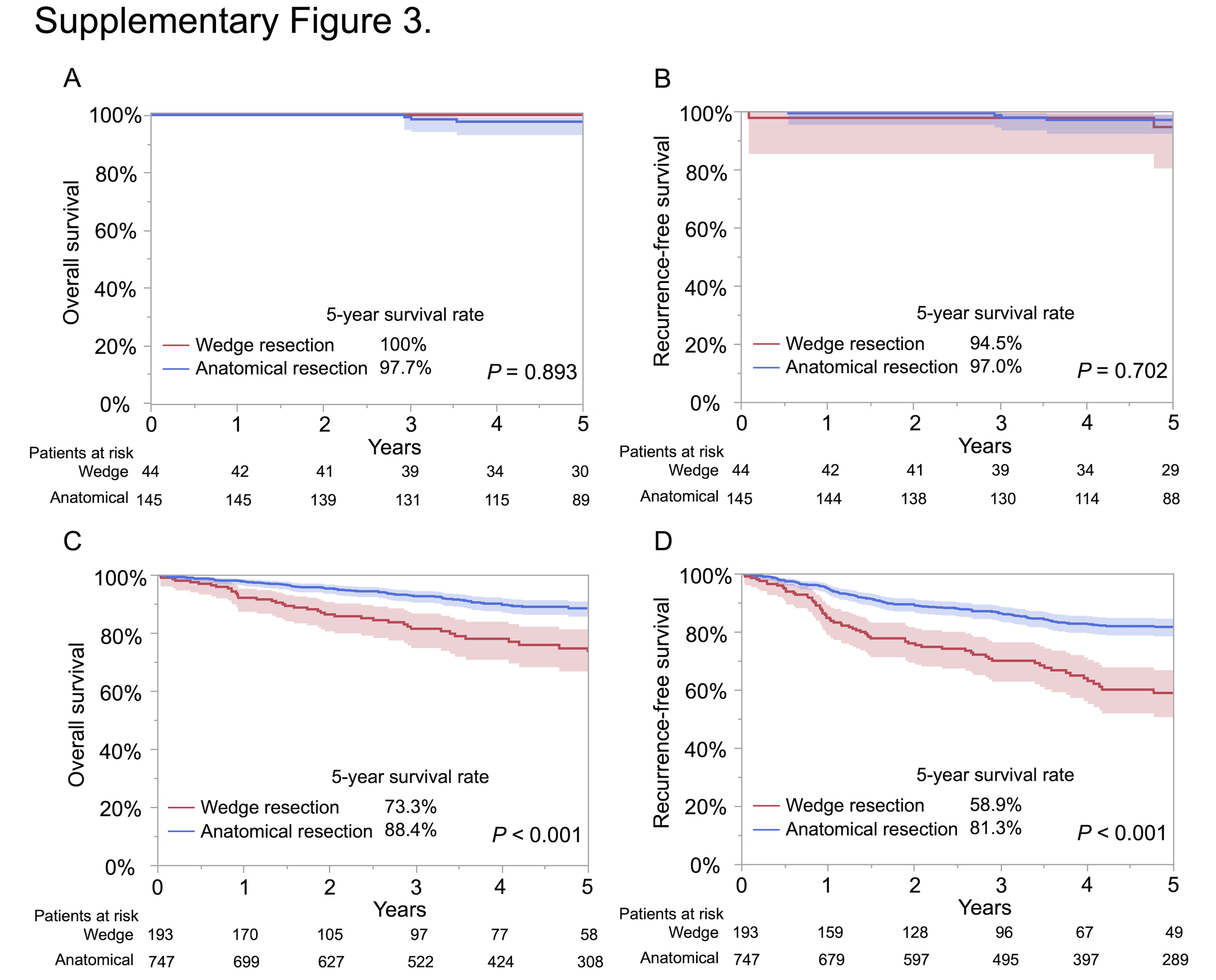

Supplement: ivac285_Supplementary_Data [file ivac285_supplementary_data.zip › Supple/Supplementary Figure 3.tiff]

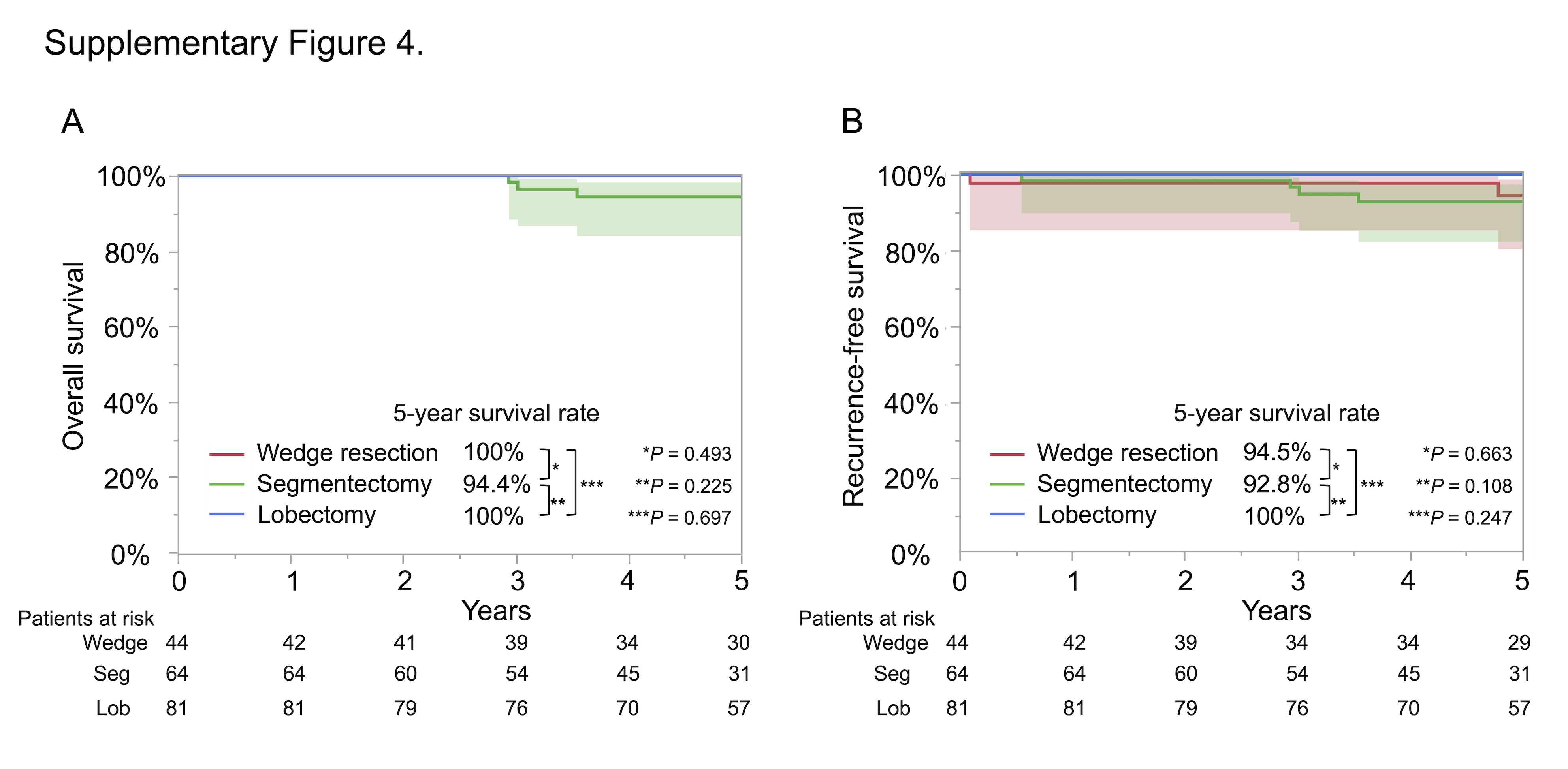

Supplement: ivac285_Supplementary_Data [file ivac285_supplementary_data.zip › Supple/Supplementary Figure 4.tiff]

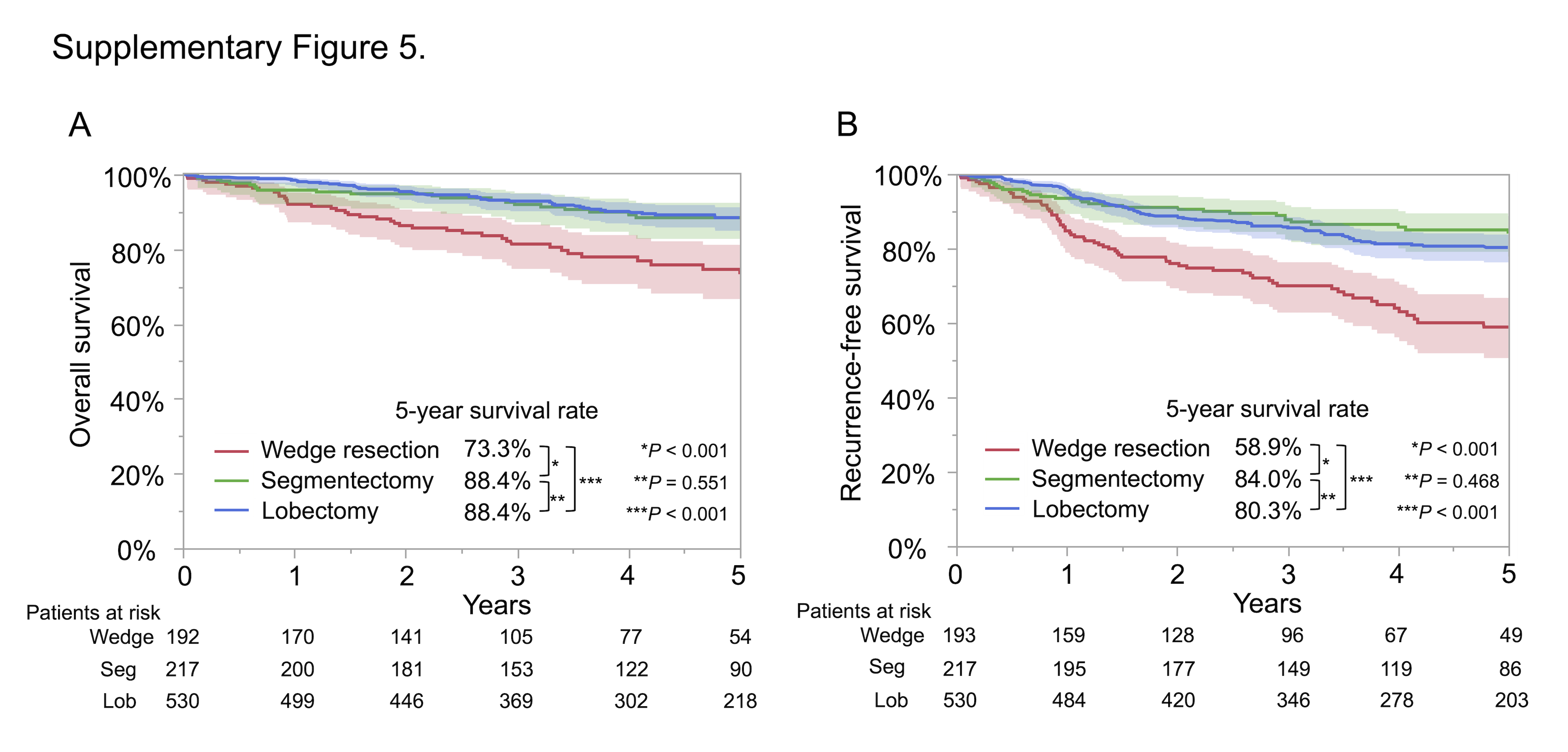

Supplement: ivac285_Supplementary_Data [file ivac285_supplementary_data.zip › Supple/Supplementary Figure 5.tiff]
